# “Synergistic Effects of Nutritional, Cognitive, and Cardiac Rehabilitation Parameters on Prognosis in Patients with Systolic Heart Failure”

**DOI:** 10.31662/jmaj.2026-0022

**Published:** 2026-08-31

**Authors:** Rintaro Furuoka, Takahiro Doi, Satoshi Kimura, Kosuke Uruno, Ryosuke Hatano, Ken Masuda, Kaoru Komuro, Hiroyuki Iwano, Daigo Nagahara, Satoshi Yuda

**Affiliations:** 1 Department of Cardiology, Teine Keijinkai Hospital, Sapporo, Hokkaido, Japan

**Keywords:** cardiac rehabilitation, cognitive dysfunction, heart failure, malnutrition, prognosis

## Abstract

**Introduction:**

It is well known that cardiac rehabilitation improves the prognosis of patients with heart failure (HF). However, there are few reports on the impact of cardiac rehabilitation interventions on the prognosis of patients with HF, malnutrition, and cognitive impairment.

**Methods:**

In 807 consecutive patients with HF and left ventricular ejection fraction <50%, the Functional Independence Measure (FIM) was used to assess the effectiveness of cardiac rehabilitation. Nutritional status and cognitive function were also assessed in all of the patients. The patients were followed up for 26.9 months with a primary endpoint of lethal cardiac events (CEs).

**Results:**

During the follow-up period, CEs were documented in 303 patients with HF. The group of patients with CEs had a lower serum cholinesterase (ChE) level, a lower Mini-Mental State Examination (MMSE) score, and lower motor and total FIM scores than the group of patients without CEs. In overall multivariate analysis, serum ChE level, MMSE score, and total FIM score were shown to be significant prognostic determinants. Receiver operating characteristic analyses revealed the cut-off values for serum ChE level, MMSE score, and total FIM score for identifying patients with HF at high risk. Patients with HF who had serum ChE <232U/L, an MMSE score <26, and a total FIM score <114 showed a significantly higher CE rate than the other patients.

**Conclusions:**

The results suggested that the addition of cardiac rehabilitation parameters to the nutritional and cognitive status of patients with HF is synergically useful for risk stratification in predicting prognosis.

## Introduction

Sarcopenia is defined as an overall decline in physiologic reserve capacity in the elderly population, and it is attributed to a multitude of factors including aging, low activity levels, inadequate nutrition, and disease in patients with heart failure (HF) ^(1)^. In the event of HF development, the patient is compelled to rest and is likely to be less active, given the observed indications of reduced endurance and muscle weakness. This condition increases the patient’s vulnerability to stress and tendency to require care. The components of sarcopenia include loss of strength and muscle mass, decreased activity, poor nutrition, and cognitive decline, each forming a vicious cycle ^(2), (3)^. In addition, the concept of cognitive dysfunction-related frailty has been proposed as a condition in which cognitive decline and physical weakness occur concurrently and share a common pathophysiological basis as sarcopenia progresses ^(4)^.

Although HF, sarcopenia, and cognitive dysfunction may appear to be independent pathologies, they are based on a common foundation of undernutrition, inflammation, and neuroendocrine abnormalities that interact to form a vicious cycle ^(5), (6), (7)^. In order to break this vicious cycle, appropriate interventions should be implemented at the stage of non‐sarcopenia and mild cognitive impairment (MCI), which are still considered reversible.

In addition to optimal medical therapy for HF, multifactorial interventions such as aerobic exercise therapy, nutritional therapy including high protein intake, and cognitive training have been shown to improve physical and cognitive functions ^(8), (9)^.

Since the number of very old patients with HF is expected to increase, it is important to detect HF in the pre-failure and MCI stages and to provide appropriate exercise and nutritional therapy in addition to drug treatment for HF in order to maintain physical and cognitive functions. The maintenance of physical and cognitive functions is of great significance ^(10), (11)^.

Recently, there have been suggestions and reports of correlations between malnutrition and cognitive and motor function. The mechanisms underlying these correlations have been partially elucidated ^(12)^. However, there are few reports on patients with cardiovascular disease and HF.

The aim of this study was to determine the incremental prognostic values for risk stratification of patients with HF and reduced left ventricular (LV) ejection fraction (LVEF), with a focus on the prognostic interactions of low nutrition, Mini-Mental State Examination (MMSE) scores to assess cognitive function, and cardiac rehabilitation function assessment.

## Materials and Methods

### Study participants and data collection

A total of 807 consecutive patients with symptomatic HF and echocardiographic LVEF below 50% were retrospectively enrolled from the Teine Keijinkai Hospital HF database between April 2012 and March 2023. The retrospective study’s enrollment criteria comprised symptomatic HF cases that required hospitalization and exhibited LVEF below 50%. Patients whose MMSE scores could not be accurately quantified due to significant cognitive decline and patients whose Functional Independence Measure (FIM) scores could not be accurately quantified due to physical decline were excluded. Patients with malignant tumors that had a clearly poor prognosis and those who refused resuscitative treatment, patients with noncardiac disease, such as severe emphysema or cirrhosis, whose prognosis was expected to be less than one year, and patients under 20 years of age were excluded. The methodology employed for the selection of patients for this study is illustrated in Figure 1. The study population included 617 male participants (76.4%) and 190 female participants (23.6%). The mean age of the recruited patients was 69.3 ± 11.5 years, and the mean LVEF was 27.2 ± 9.8%. The diagnosis of HF was made by experienced cardiologists with reference to the Framingham criteria. The criteria for diagnosis included typical symptoms such as distended jugular veins, peripheral edema, pulmonary rales, S3 or S4 gallop, and tachycardia. To confirm the final diagnosis of HF and to exclude conditions with similar symptoms and signs, chest X-rays and two-dimensional transthoracic echocardiography were conducted. Ischemic and non-ischemic etiologies were distinguished by electrocardiography, echocardiography, cardiac scintigraphy, or a combination of those, as necessary, along with the acquisition of coronary artery information obtained through computed tomography, magnetic resonance imaging, and invasive coronary angiography, in addition to a prior history of myocardial infarction and/or coronary revascularization. Blood tests for hemoglobin, creatinine, serum sodium, serum cholinesterase, and brain natriuretic peptide (BNP) were performed prior to discharge from the hospital after compensated HF and optimal medical therapy. Total protein, albumin, and lipid profiles were measured simultaneously. Renal function was also evaluated by estimated glomerular filtration rate estimated glomerular filtration rate using the standard formula. Baseline Geriatric Nutritional Risk Index was calculated from serum albumin and body mass index using the following formula: GNRI = 14.89 × serum albumin (g/dL) + 41.7 × present body weight (kg) / [height ^(2)^ (m^2^) × 22]

**Figure 1.**
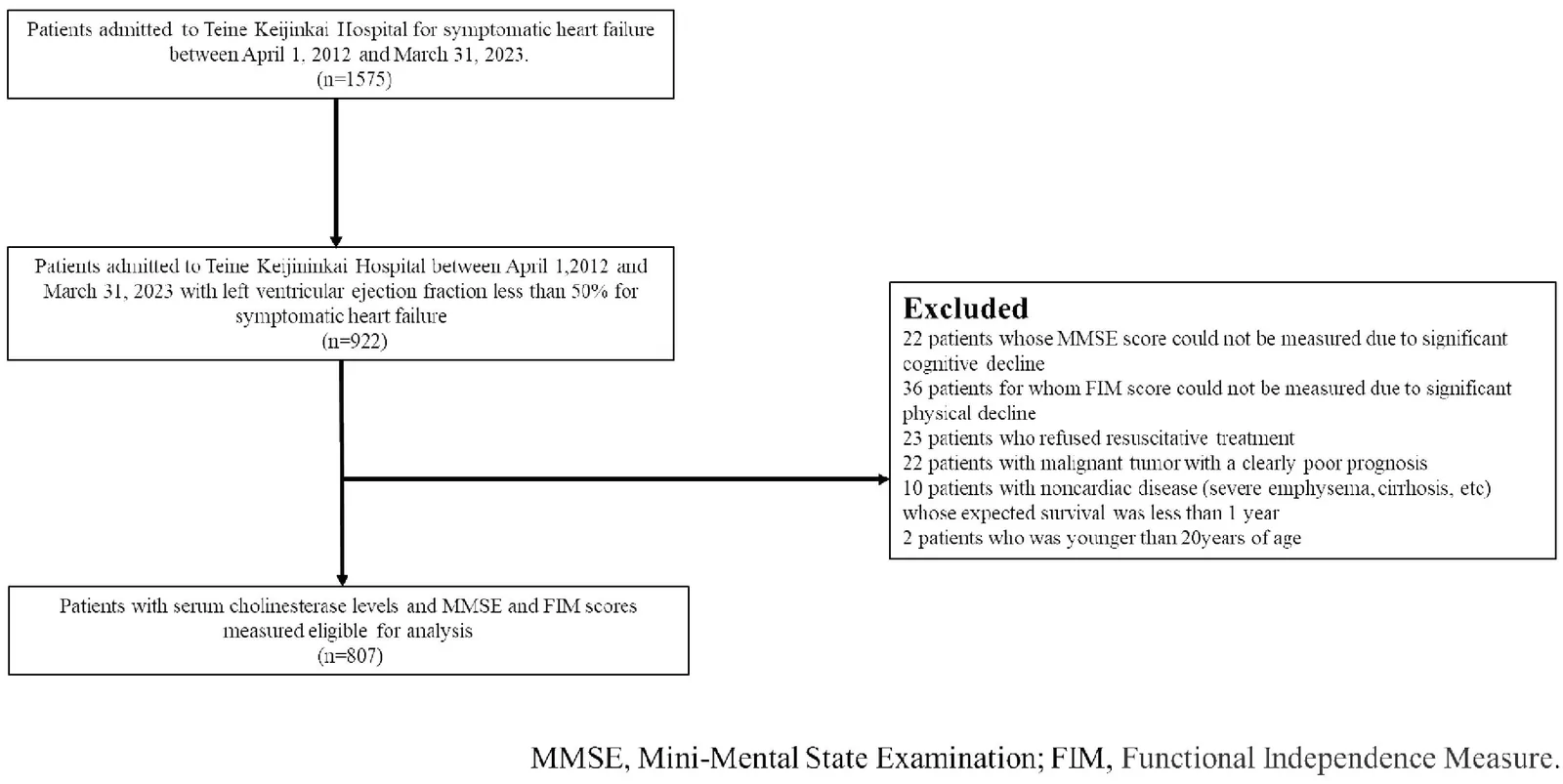
Flowchart of the present study.

**Figure 2.**
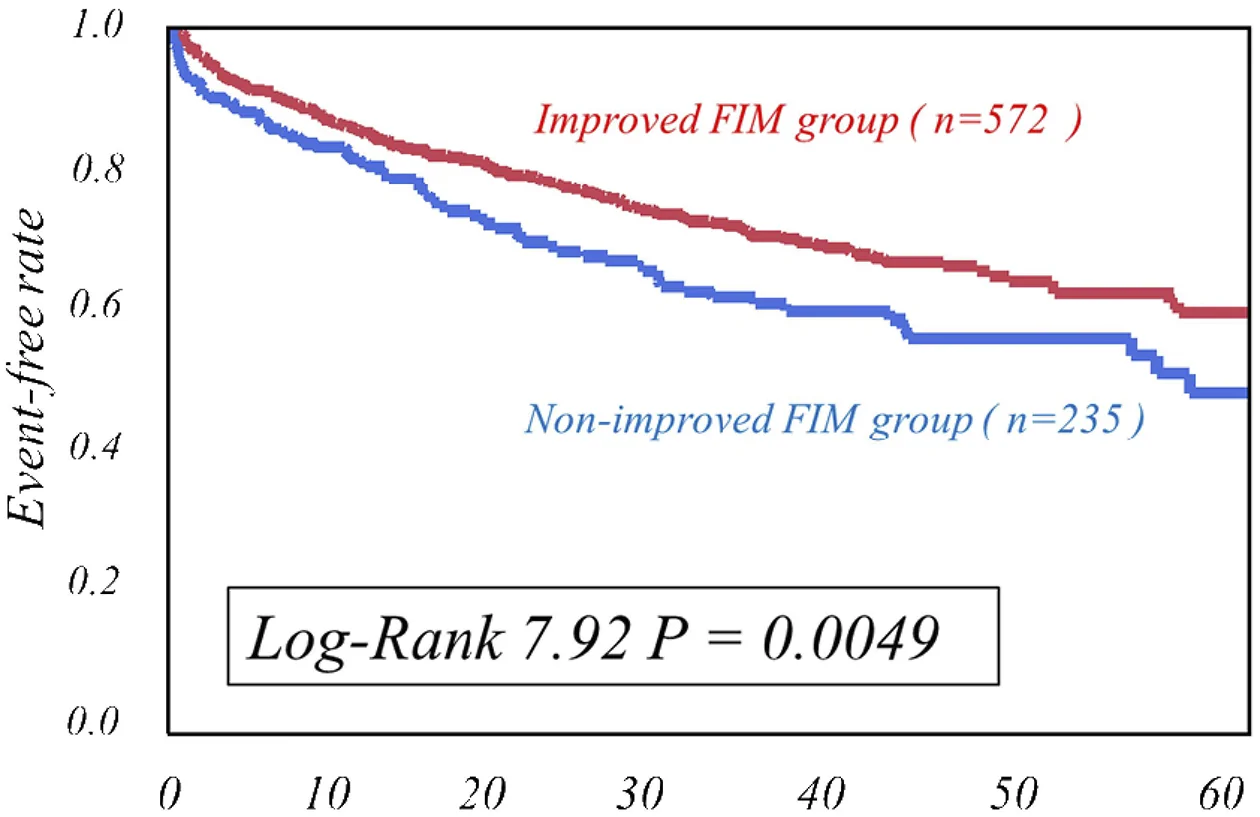
In the Kaplan–Meier event-free survival curves, categorization based on changes in total FIM score during hospitalization demonstrated clear separation between the Improved FIM group and the Non-improved FIM group. Patients who exhibited any increase in total FIM score showed higher event-free survival rates, whereas those whose scores remained unchanged or decreased experienced markedly lower event-free survival. FIM: functional independence measure.

### Measurement of cognitive function

The MMSE was used to evaluate and assess dementia due to its brief testing time (10-15 minutes) and ease of administration without special equipment ^(13)^. The MMSE is an 11-item cognitive function test with a total score of 30. The test includes assessments of time perception, place perception, immediate and delayed recall of three words, calculation, object naming, sentence recitation, three levels of verbal commands, written commands, written text, and graphic imitation. An MMSE score of 23 or below may indicate dementia, while a score of 27 or below may indicate MCI. Experienced staff of the hospital rehabilitation department conducted the MMSE without having received patient information or clinical data on outcomes and prognosis.

### Assessment of the effectiveness of cardiac rehabilitation

The FIM is a tool for evaluating activities of daily living (ADLs). It is distinguished by its ease of application because it relies solely on the assessment of present movements and cognitive abilities, obviating the need for medical knowledge ^(14), (15)^. The FIM assesses the state of ADLs from the overall total score on a scale of 7 to 1 for a total of 18 items, including 13 items for actual movements (motor items) and 5 items for cognitive functions (cognitive items). Since each of the 18 items is scored on a scale of 7, a wide range of scores from 18 to 126 is possible. The system is capable of providing a wide range of ratings from 18 to 126 points.

FIM assessment is widely regarded as highly dependable for ADLs. Its detailed grading of activities that can be undertaken means that it is particularly accurate in determining the state of an individual’s ADLs. It is widely believed that the use of repeated assessments can reveal even minor improvements in an individual’s condition, making it an effective tool for evaluating the effectiveness of rehabilitation strategies and other interventions. FIM assessment enables informed decisions regarding treatment and management policies.

For the present analysis, the total FIM score obtained at hospital admission was defined as the FIM at baseline, and the score obtained at the time of discharge was defined as the FIM at follow-up. To evaluate functional recovery during the hospitalization period, patients were further categorized into two groups based on changes in the total FIM score from baseline to follow-up. The Improved FIM group was defined as patients who demonstrated any increase in total FIM score, whereas the non-improved FIM group consisted of patients whose total FIM score remained unchanged or decreased.

### Echocardiographic assessment

Using an ultrasound system equipped with a 2.5 MHz variable-frequency transducer, the echocardiographer conducted a two-dimensional echocardiographic scan from the paracentral long-axis image, along with apical 4-, 3- and 2-chamber images, with the patient in the left lateral decubitus position. Echocardiographic parameters were measured under stable conditions prior to discharge. The parameters included left atrial diameter (mm), LV end-diastolic diameter (mm), LVEF (%) calculated using the biplane modified Simpson method, LV end-diastolic volume (mL), LV end-systolic volume (mL), and septal E/e ^(16)^.

### Follow-up protocol

Patients with HF in our HF database received regular outpatient follow-up from cardiologists for at least one year, provided they survived. The primary endpoint for this study was the analysis of fatal cardiac events (CEs), encompassing sudden cardiac death, death attributed to progressive pump failure, fatal sustained ventricular tachyarrhythmias, and appropriate implantable cardioverter defibrillator therapy for fatal ventricular arrhythmias. Through retrospective analysis of medical records and outcome assessments, the clinical courses and outcomes were confirmed. Sudden cardiac death was defined as an observed cardiac arrest and death within one hour of acute onset of symptoms or unexpected death of a patient who had previously been healthy within 24 hours. Our Ethics Committee approved this study for entry into our database and use of data for clinical research purposes in accordance with the principles of the Declaration of Helsinki. The approval number 2-023226-00 was assigned to the study. Because of its retrospective and observational design, the study was exempted from obtaining written informed consent from each participant.

### Statistical analysis

Statistical data are presented as means ± standard deviation. A comparison of mean values was conducted between the two groups through unpaired t-tests, while χ^2^ tests were used to compare categorical variables. Significant key parameters selected in the multivariate analysis of this study were used to generate time-dependent cumulative event-free survival curves through Kaplan-Meier analysis. Log-rank tests were used when necessary to compare the curves. The Cox proportional hazard model was used to evaluate the hazard ratios and 95% confidence intervals (CIs) for the significant variables, with consideration of the statistically relevant number of significant variables identified in the univariable analysis, subject to the number of CEs. Receiver operating characteristic (ROC) analysis was used to determine the preferred cut-off values for independent significant parameters. Analyses were conducted using SAS for Windows, version 9.4 (SAS Institute, Cary, North Carolina, USA), with p values less than 0.05 considered significant. Moreover, Mathematica software (version 14.0, Wolfram Research Inc., Champaign, IL, USA) was used to analyze additional Net Reclassification Index (NRI) ^(17)^ to prove the incrementality of the area under the curve ^(18)^ and test the complementary log-log plot ^(19)^ to confirm proportional hazards.

## Results

In 303 HF patients (37.5%), CEs recorded during a mean follow-up period of 26.8 months included 54 sudden cardiac deaths, 215 cardiac deaths due to refractory and progressive pump failure, 12 fatal ventricular tachyarrhythmias, and 22 appropriate implantable cardioverter defibrillator treatments for fatal ventricular tachyarrhythmias.

Patients with HF and CEs were older than patients with HF without CEs, and patients with HF and CEs had a higher New York Heart Association functional class, lower estimated glomerular filtration rate (36.9 ± 25.5 vs 48.7 ± 23.5 mL/min/1.73m^2^, p < 0.001), lower hemoglobin level (11.7 ± 2.2 vs 12.9 ± 2.3 g/dL, p < 0.001) and higher BNP level (1077.4 ± 1431.1 vs 692.9 ± 895.3 pg/mL, p < 0.001) than patients with HF without CEs (Table 1a). Patients with HF and CEs had a lower level of serum cholinesterase (209.7 ± 68.6 vs 265.8 ± 80.9 U/L, p < 0.001), lower MMSE score (23.4 ± 4.8 vs 28.8 ± 2.7, p < 0.001), lower total FIM at baseline (72.5 ± 30.3 vs 90.8 ± 31.4, p < .001) and lower total FIM at follow-up (74.5 ± 36.7 vs 113.5 ± 17.3, p < 0.001) than patients with HF without CEs (Table 1b).

**Table 1(a).** Comparison of Clinical Data and Medication Between Groups With and Without Cardiac Events.

	Cardiac events group (n = 303)	Non-cardiac events group (n = 504)	p-value
Age (years)	72.2 ± 10.1	67.5 ± 12.0	p < 0.0001
Sex (male/female)	230/73	387/117	p = 0.7761
NYHA (Ⅰ/Ⅱ/Ⅲ/Ⅳ)	194/58/39/12	494/7/3/0	p < 0.0001
Body mass index (kg/m^2^)	22.2 ± 4.1	23.7 ± 4.2	p < 0.0001
Past history			
Hypertension	107 (35.3%)	249 (49.4%)	p = 0.0021
Diabetes mellitus	117 (38.6%)	208 (41.2%)	p = 0.0921
Dyslipidemia	126 (41.5%)	232 (46.0%)	p = 0.2135
Atrial fibrillation	118 (38.9%)	177 (35.1%)	p = 0.2712
Ventricular tachycardia / Ventricular fibrillation	81 (26.7%)	91 (18.1%)	p = 0.0034
Etiology			
Ischemic	157 (51.8%)	236 (46.8%)	p = 0.1689
Device implantation			
ICD implantation	77 (25.4%)	93 (18.4% )	p = 0.0190
CRT implantation	53 (17.4%)	80 (15.8% )	p = 0.5411
TAVI	6 (1.9%)	6 (1.2%)	p = 0.3701
MitraClip implantation	2 (0.6%)	9 (1.8%)	p = 0.1529
Laboratory data			
Hemoglobin (g/dL)	11.7 ± 2.2	12.9 ± 2.3	p < 0.0001
eGFR (mL/min/1.73 m^2^)	36.9 ± 25.5	48.7 ± 23.5	p < 0.0001
BNP (pg/mL)	1077.4 ± 1431.1	692.9 ± 895.3	p < 0.0001
Medications			
ACE-I/ARBs	162 (53.4%)	392 (58.1%)	p = 0.2371
ARNI	4 (1.3%)	72 (14.2%)	p < 0.0001
β blockers	274 (90.4%)	481 (95.4%)	p = 0.0793
Loop diuretics	235 (77.5%)	372 (73.8%)	p = 0.0001
Anti-aldosterone agents	140 (46.2%)	301 (59.7%)	p < 0.0001
SGLT inhibitors	7 (2.3%)	110 (21.8%)	p < 0.0001
Anti-vasopressin agents	129 (42.5%)	198 (39.2%)	p = 0.3158
Amiodarone	112 (36.9%)	153 (30.3%)	p = 0.0691
Statins	145 (47.8%)	299 (59.3%)	p = 0.0001

Values are shown as means ± one standard deviation. ACE-I: angiotensin-converting enzyme inhibitor; ARB: angiotensin receptor blocker; ARNI: angiotensin receptor-neprilysin inhibitor; CRT: cardiac resynchronization therapy; eGFR: estimated glomerular filtration rate; ICD: implantable cardioverter-defibrillator; ns: not significant; NYHA: New York Heart Association functional classification; SGLT: sodium-glucose cotransporter; TAVI: transcatheter aortic valve implantation.

**Table 1(b).** Comparison of Two-Dimensional Echocardiographic Parameters and Parameters Related to Nutrition, Cognitive Function and Cardiac Rehabilitation Between Groups With and Without Cardiac Events.

	Cardiac events group (n = 303)	Non-cardiac events group (n = 504)	p-value
M-mode			
LVDd (mm)	56.7 ± 9.7	56.8 ± 8.6	p = 0.8835
LVDs (mm)	48.8 ± 11.7	48.6 ± 10.1	p = 0.8562
LAD (mm)	47.6 ± 8.6	46.0 ± 8.4	p = 0.0074
Modified Simpson method			
LVEF (%)	27.2 ± 10.1	27.2 ± 9.6	p = 0.9822
EDV (mL)	154.5 ± 77.6	147.8 ± 54.3	p = 0.1542
ESV (mL)	115.7 ± 69.9	110.0 ± 49.1	p = 0.1856
Tissue Doppler method			
Septal E/e’	18.6 ± 9.2	16.2 ± 7.1	p < 0.0001
Laboratory data related to nutrition			
Total protein (g/dL)	6.7 ±V 0.7	6.9 ± 0.7	p < 0.0001
Albumin (g/dL)	3.5 ± 0.6	3.8 ± 0.5	p < 0.0001
Total cholesterol (mg/dL)	157.8 ± 37.0	170.5 ± 43.1	p < 0.0001
High-density lipoprotein (mg/dL)	44.9 ± 15.6	49.2 ± 18.8	p = 0.0021
Low-density lipoprotein (mg/dL)	88.1 ± 29.8	95.7 ± 34,1	p = 0.0026
Serum triglycerides (mg/dL)	102.8 ± 54.1	120.2 ± 68.4	p < 0.0001
Serum cholinesterase (U/L)	209.7 ± 68.6	265.8 ± 80.9	p < 0.0001
Malnutrition parameter			
Geriatric Nutrition Risk Index	95.1 ± 12,5	101.6 ± 11.9	p < 0.0001
Cognitive parameter
Mini-Mental State Examination	23.4 ± 4.8	28.8 ± 2.7	p < 0.0001
Rehabilitation parameters			
Total FIM at baseline	72.5 ± 30.3	90.8 ± 31.4	p < 0.0001
Total FIM at follow-up	74.5 ± 36.7	113.5 ± 17.3	p < 0.0001

Values are shown as means ± one standard deviation. GNRI (Geriatric Nutrition Risk Index) = 14.89 × Alb+ 41.7 × (current body weight)/(standard body weight). Dct: left ventricular deceleration time; EDV: left ventricular end-diastolic volume; ESV: left ventricular end-systolic volume; FIM: Functional Independence Measure; IVSTd: end-diastolic interventricular septal wall thickness; LAD: left atrial diameter; LV: left ventricular; LVDd: end-systolic left ventricular diameter; LVEF: left ventricular ejection fraction; ns: no significance; PWTd: end-diastolic posterior wall thickness.

To further explore the prognostic relevance of functional recovery, changes in total FIM score from baseline to follow-up were evaluated. Patients were classified into two groups according to whether any improvement in total FIM score was observed during the hospitalization period. A total of 572 patients (70.8%) demonstrated an increase in FIM score, whereas 235 patients (29.2%) showed no improvement. Patients with FIM improvement exhibited significantly higher CE-free survival rates compared with those without improvement.

Among the significant univariate variables (Table 2), serum cholinesterase, MMSE score, and total FIM at follow-up, together with age, NYHA functional class, and BNP level, were shown to be significant independent prognostic factors in multivariate Cox analysis with the following hazard ratios. Hazard ratios and 95% CIs (p values) for serum cholinesterase, MMSE score, and total FIM at follow-up were 0.995, 0.992-0.997 (p < 0.001), 0.966, 0.938-0.996 (p = 0.02), and 0.987, 0.982-0.992 (p < 0.001), respectively.

**Table 2.** Results of Univariate and Multivariate Analyses.

	Univariate analysis					Multivariate Cox proportional-hazards model analysis				
			95% CI					95% CI		
	χ^2^	Hazard ratio	Lower	Upper	*p*-value	χ^2^	Hazard ratio	Lower	Upper	*p*-value
Age (years)	62.5	1.044	1.032	1.057	< 0.0001	18.1	1.031	1.016	1.047	< 0.0001
NYHA functional class	153	2.476	2.189	2.772	< 0.0001	39.4	1.754	1.486	2.058	< 0.0001
Hemoglobin level	57.4	0.817	0.774	0.862	< 0.0001	2.01	0.949	0.884	1.019	0.1143
eGFR	57.5	0.981	0.976	0.986	< 0.0001	0.64	0.997	0.991	1.003	0.4244
BNP	32.4	1.000	1.000	1.001	< 0.0001	5.11	1.000	1.000	1.001	0.0219
ARNI	12.8	0.243	0.075	0.573	< 0.0001	0.35	0.694	0.157	2.120	0.5538
SGLT inhibitors	15.8	0.292	0.124	0.577	< 0.0001	0.42	0.746	0.268	1.699	0.5129
Septal E/e’	20.9	1.035	1.020	1.050	< 0.0001	0.76	0.993	0.978	1.008	0.3879
Serum cholinesterase	128	0.991	0.989	0.992	< 0.0001	17.1	0.995	0.992	0.997	<0.0001
MMSE	161	0.868	0.852	0.885	< 0.0001	4.71	0.966	0.938	0.996	0.0238
Total FIM at follow-up	215	0.997	0.974	0.980	< 0.0001	25.3	0.987	0.982	0.992	< 0.0001

ARNI: angiotensin receptor neprilysin inhibitor; CI: confidence interval; FIM: Functional Independence Measure; GFR: glomerular filtration rate; MMSE: Mini-Mental State Examination; NYHA: New York Heart Association; SGLT: sodium glucose cotransporter.

The cut-off values for serum cholinesterase, MMSE, and total FIM at follow-up, which define the prognosis of patients with HF, were calculated by ROC analysis, and they were 232 U/L, 26, and 114, respectively (Figure 3a). Patients with HF who had serum cholinesterase of less than 232 U/L, an MMSE score of less than 26, or total FIM at follow-up of less than 114 had significantly lower CE-free rates than the other patients with HF (Figure 3b). Combining two of the three parameters (serum cholinesterase, MMSE score, and total FIM at follow-up) more clearly discriminated patients with HF, allowing three risk categories to be more clearly identified: low, intermediate, and high risks (Figure 4). A cumulative score of the three parameters (i.e, serum cholinesterase <232 U/L, MMSE score <26 and total FIM at follow-up <114) was shown to increase the risk of CEs in patients with HF (Figure 5).

**Figure 3.**
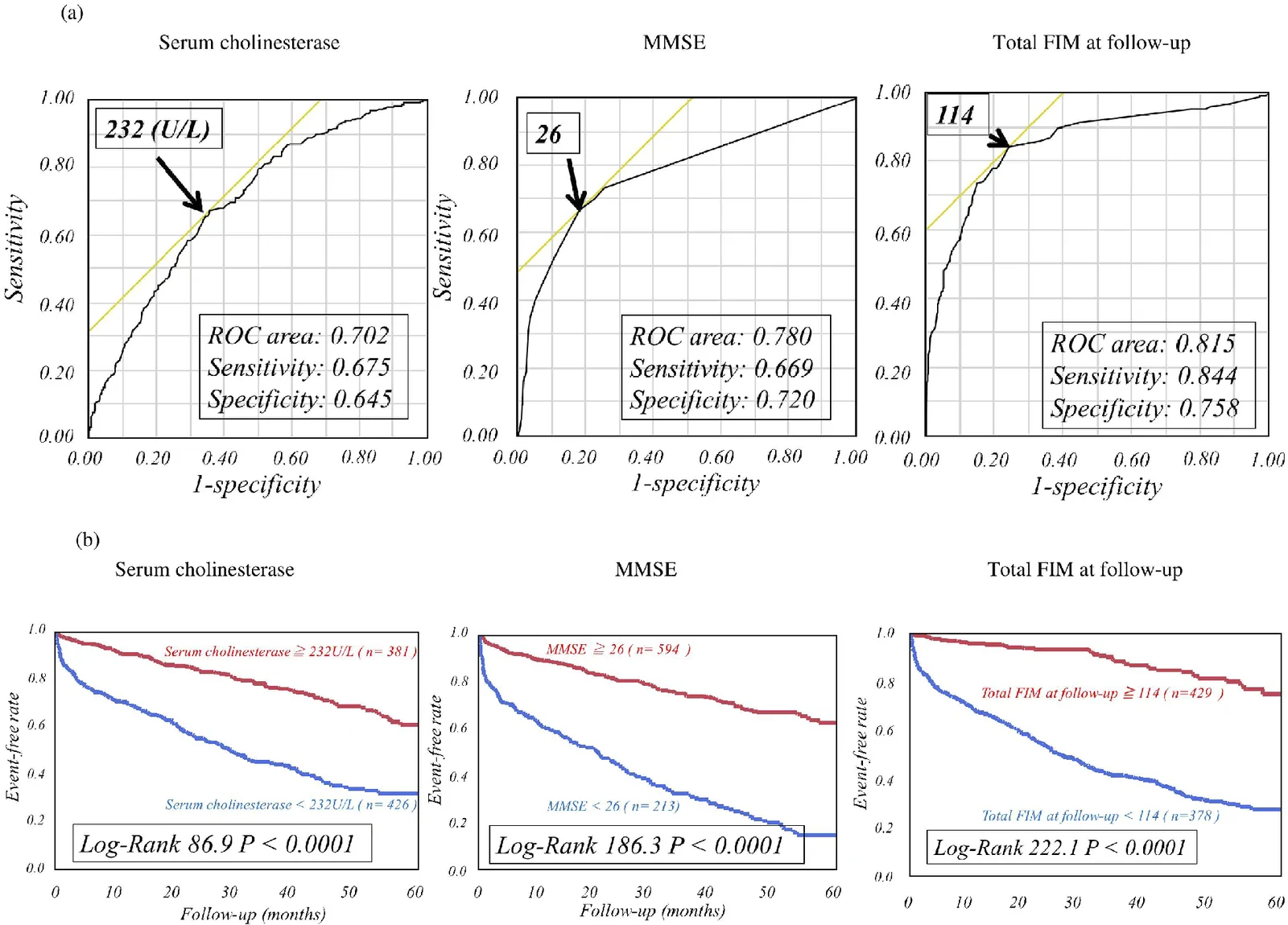
a: ROC analysis yielded cut-off values of 232 U/L, 26, and 114 for serum cholinesterase, MMSE score, and total FIM score, respectively. b: By means of these cut-off values, Kaplan-Meier event-free curves demonstrate evident differentiation between the categories of high-risk patients and low-risk patients. FIM: functional independence measure; MMSE: mini-mental state examination; ROC: receiver operating characteristic.

**Figure 4.**
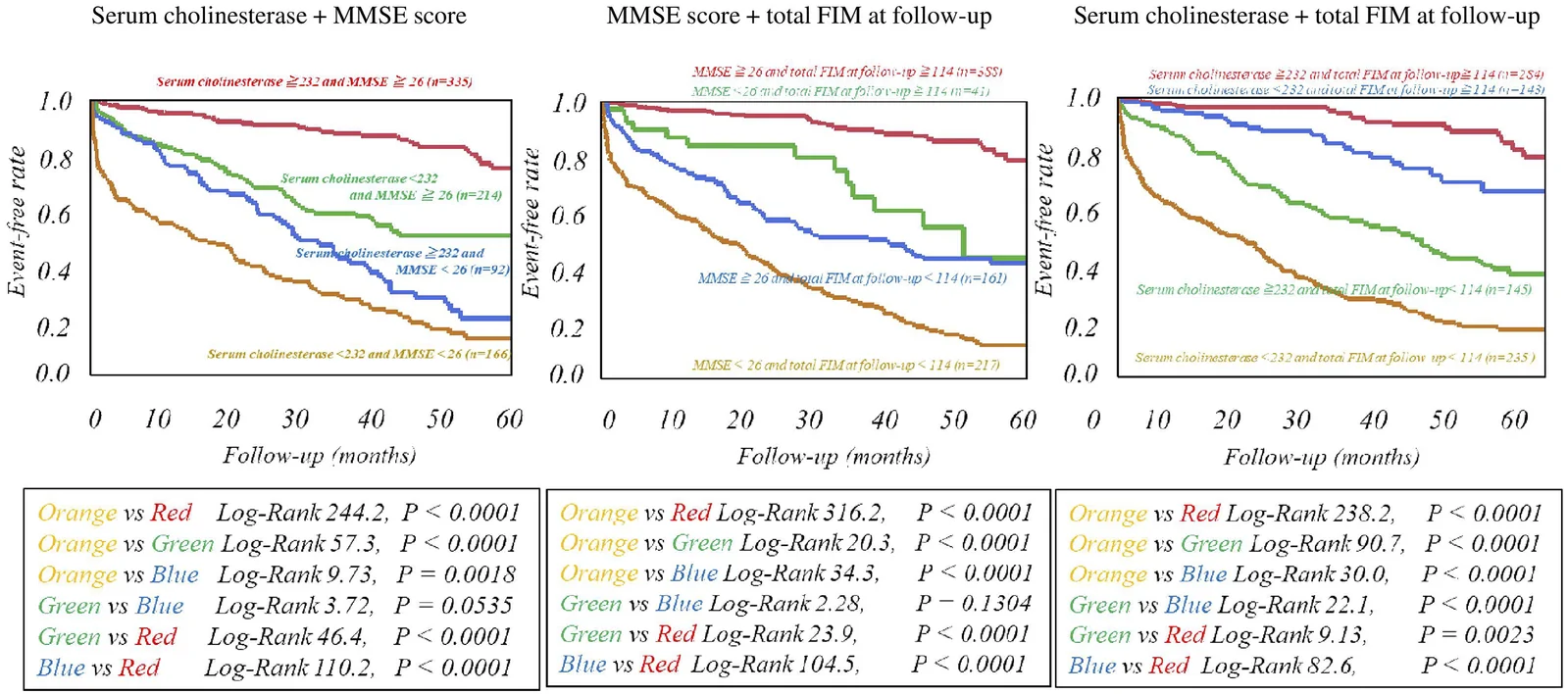
Kaplan-Meier event-free survival curves were used to clearly classify patients into low, intermediate, and high-risk groups using two of the three prognostic variables, including serum cholinesterase, MMSE score, and total FIM score. FIM: functional independence measure; MMSE: mini-mental state examination.

**Figure 5.**
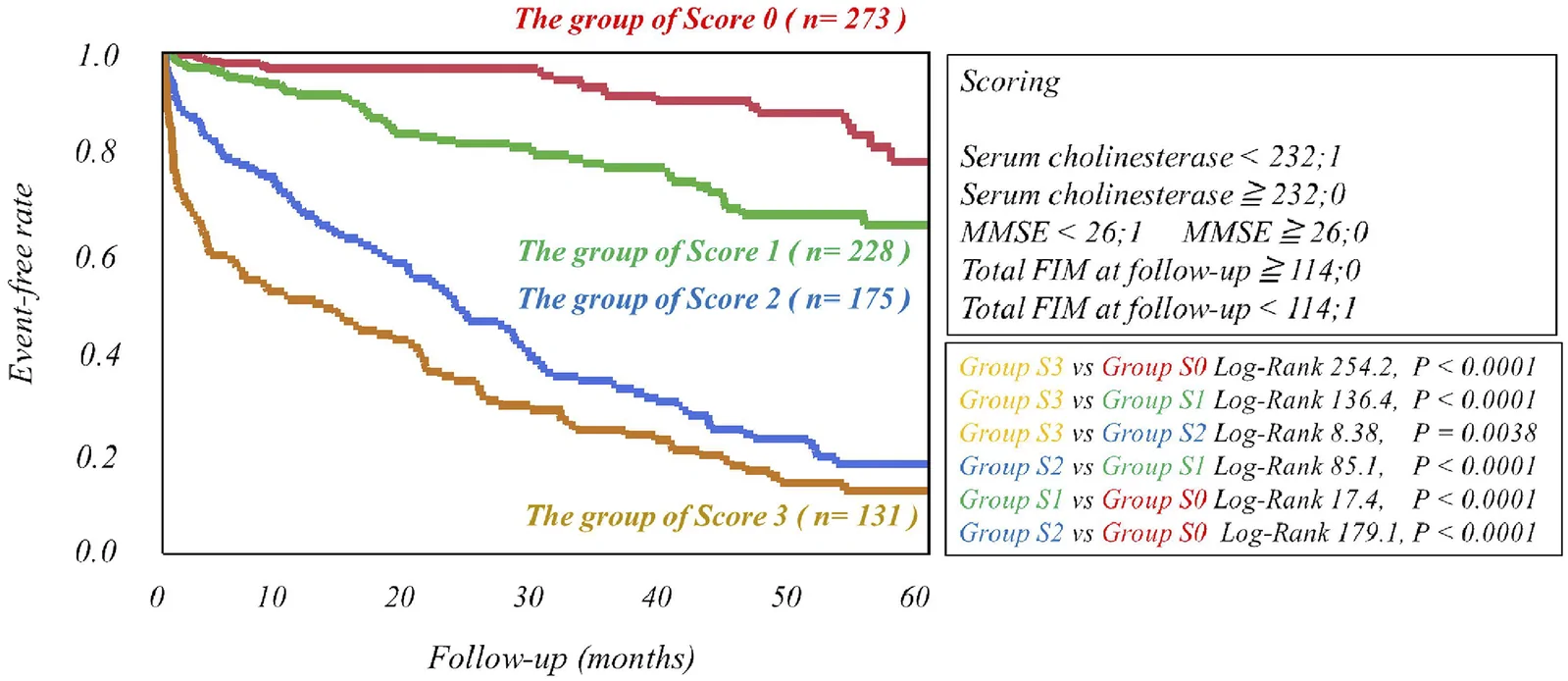
In the Kaplan-Meier event-free survival curves, accumulation of the three abnormal parameters (i.e., serum cholinesterase <232 U/L, MMSE score <26, and total FIM score <114) was associated with a synergistic reduction in event-free survival. The number of abnormal parameters is indicated by the score category (0-3). FIM: functional independence measure; MMSE: mini-mental state examination.

The addition of prognostic parameters, as assessed using the Net Reclassification Index (NRI), improved the probability of predicting non-CEs. While there was no significant difference in predicting CEs between Model 1 and Model 2 or between Model 3 and Model 4 when the NRI was used, there was a significant difference when CE and non-CE predictions were combined, indicating stratified risk of HF in Supplementary Table 1. Supplementary Table 2 suggested that the area under the curve increases additively when serum cholinesterase level, MMSE score, and total FIM at follow-up score are combined. Supplementary Table 3 demonstrated confirmation of the proportional hazard model.

## Discussion

The total FIM score at follow-up was found to predict prognosis independently, as did serum cholinesterase levels and MMSE score, which have traditionally been used as prognostic parameters in patients with HF. Furthermore, when combined with measures of nutritional status and cognitive function, the findings indicated that the parameters for assessing cardiac rehabilitation effectiveness may significantly and cumulatively stratify the risk of patients with HF. In addition to these findings, stratification based on changes in total FIM score during hospitalization also demonstrated prognostic relevance, as patients who exhibited improvement in FIM score showed higher CE-free survival rates than those without improvement. However, the degree of risk discrimination achieved by FIM improvement alone was less pronounced than that obtained using the total FIM score at follow-up together with serum cholinesterase level and MMSE score.

In cardiovascular medicine, exercise therapy is considered to be a vital component of secondary prevention, particularly in HF management. Additionally, exercise therapy has a central role in cardiac rehabilitation programs ^(20), (21)^. Nutritional status is one of the factors influencing program planning, load and goal setting, and the effectiveness of exercise therapy. Healthcare professionals, including doctors, therefore need to have a detailed understanding of nutritional status and the state of skeletal muscles that reflect nutritional status ^(22), (23)^. Muscle strength and muscle mass are regarded as important components in nutritional status. Muscle strength, walking speed, and standing movements are also important components in sarcopenia, which is a loss of muscle strength and muscle mass, and in frailty, which is a loss of reserve capacity for life and living ^(24), (25)^.

Low nutrition and sarcopenia, especially in elderly patients with HF, as well as the presence of cachexia as the disease progresses, certainly worsen the prognosis of patients with HF.

This study suggests that although improvement in total FIM score during hospitalization was associated with a more favorable clinical course, the prognostic value of FIM improvement alone may be constrained by ceiling effects, particularly in patients who already exhibit relatively high functional status at baseline and therefore have limited measurable room for further improvement. In contrast, the combination of total FIM score at follow-up with serum cholinesterase level and MMSE score provided clearer and more consistent risk discrimination, suggesting that integrated assessment of nutritional, cognitive, and functional domains offers a more robust framework for prognostic stratification in HF patients.

It has been reported that patients with HF are prone to sarcopenia, a component of physical frailty, mainly due to reduced muscle mass and muscle strength caused by decreased cardiac output, and the interaction between HF and frailty has attracted attention ^(4), (26)^. On the other hand, the concept of cognitive frailty has been proposed as a condition in which cognitive decline and physical frailty occur simultaneously on a common pathophysiological basis ^(4), (27)^. HF, frailty, and cognitive dysfunction were once thought to be independent pathologies; however, they have recently been considered to be based on a common foundation of low nutrition, inflammation, and neuroendocrine abnormalities, which interact with each other to form a vicious cycle. To break the vicious circle, appropriate interventions should be provided at the stage of pre-frailty and MCI, which are still considered reversible.

Malnutrition (i.e., a deficiency in both protein and energy) is a significant risk factor for both quality of life and mortality in older adults. It has been observed that malnutrition is associated with poor prognosis, even in patients diagnosed with HF ^(28), (29)^. The European Society for Vein and Enteral Nutrition (ESPEN) guidelines on nutritional management for older individuals with dementia reference the ‘vicious cycle’ of undernourishment. Cognitive impairment, as well as other age-related geriatric syndromes, pose a risk for undernourishment in older people, and undernourishment can lead to frailty, sarcopenia, and cognitive impairment ^(30)^. A high level of evidence from the ESPEN suggests that the most effective intervention for low nutritional status compounded by cognitive decline is the provision of oral nutritional support to enhance nutritional status ^(31), (32), (33)^.

### Study limitations and future perspectives

This study has several limitations. First, it was conducted as a retrospective, observational, non-interventional cohort study at a single center, and the findings may not be fully generalizable to broader populations. Individuals with irreversible systolic HF were included, and only clinical parameters routinely documented in medical records were available for analysis.

Second, although exercise tolerance is an important prognostic indicator in HF, objective assessments such as peak oxygen consumption (VO_2_) and anaerobic threshold measured by cardiopulmonary exercise testing ^(34), (35)^ were not performed in this cohort. The Borg scale ^(36)^, which can serve as a surrogate when cardiopulmonary exercise testing is not feasible, was also not recorded. Furthermore, because of the retrospective design, no additional standardized measures of functional capacity, such as the 6-minute walk test ^(37)^, gait speed, or other exercise-related assessments, were available. As a result, the NYHA functional class was the only functional capacity indicator consistently obtainable for all patients.

Third, several validated nutritional screening tools and biochemical nutritional risk indices, including Malnutrition Universal Screening Tool, Prognostic Nutritional Index, NRI, and Controlling Nutritional Status, were not assessed in this study ^(38), (39), (40), (41)^. Similarly, other cognitive function assessments, such as the Hasegawa Dementia Scale-Revised ^(42)^, Mini-Cog ^(43)^, Montreal Cognitive Assessment ^(44)^, Dementia Assessment Sheet for Community-based Integrated Care System-21 items ^(45)^, and ABC Dementia Scale ^(46)^, were not performed.

Fourth, the analysis of changes in total FIM score was exploratory and may have been influenced by ceiling effects in patients with relatively high baseline functional status, as well as by variations in the duration and intensity of rehabilitation during hospitalization. Consequently, the prognostic implications of FIM improvement should be interpreted with caution.

Future multicenter prospective studies incorporating comprehensive assessments, including objective measures of exercise tolerance, standardized functional capacity tests, detailed nutritional evaluations, and multidimensional cognitive assessments, are warranted. Such studies may help refine the appropriate indications and timing of multidisciplinary interventions, including nutritional management, pharmacological and non-pharmacological treatment, and cardiac rehabilitation, for patients with HF, malnutrition, cognitive impairment, and frailty.

### Conclusions

This study suggested that incorporation of cardiac rehabilitation parameters into nutritional and cognitive status assessment for patients with HF is a mutually beneficial means for predicting prognosis through risk stratification.

## Article Information

### Acknowledgments

We express our gratitude to all of the physiotherapists, dietitians, pharmacists, social workers, ward and outpatient nurses, and staff who participated in the Heart Failure Multidisciplinary Conference at Teine Keijinkai Hospital (Sapporo) for their valuable assistance.

### Author Contributions

Principal investigators of this study participated in the study design, collection of patient data, data analysis, and drafting the initial version of the manuscript: Rintaro Furuoka, Takahiro Doi, Satoshi Kimura, Kosuke Uruno, Ryosuke Hatano, Ken Masuda, Kaoru Komuro, Hiroyuki Iwano, Daigo Nagahara. Supervised all parts of this study and contributed to preparation of the final manuscript: Satoshi Yuda. All authors have read and approved submission of the manuscript, and the manuscript has not been published and is not being considered for publication elsewhere in whole or part in any language except as an abstract. All authors meet the 4 ICMJE criteria for authorship below.

1. All authors have made significant contributions to the conception or design of the work or to the acquisition, analysis and interpretation of data from the work.

2. All authors have drafted or critically revised their work because of its important intellectual content.

3. All authors gave final approval for the published version.

4. All authors agreed to take responsibility for all aspects of their work to ensure that any questions about the accuracy or completeness of their work are properly investigated and resolved.

### Conflicts of Interest

None

### Approval Code Issued by the Institutional Review Board (IRB) and the Name of the Institution(s) that Granted the Approval.

The approval number 2-023226-00 was assigned to the study. Because of its retrospective and observational design, the study was exempted from obtaining written informed consent from each participant.

### Data Availability

The deidentified participant data will be shared upon request. Please contact the corresponding author directly to request data sharing. The entire dataset used will be available, including the study protocol. The data will be available to anyone. Any analysis of the data will be approved and the data will be shared as an anonymized Excel file via email.

## Supplement

Supplementary Material Supplementary Table 1.
The incorporation of prognostic parameters and the utilization of the NRI enhanced the probability of forecasting non-CEs. Whilst there was no notable discrepancy in the prediction of CEs between Model 1 and Model 2, or between Model 3 and Model 4 when the NRI was employed, a significant divergence was observed when CE and non-CE predictions were integrated, indicating a stratified risk of HF.
Supplementary Table 2.
When serum cholinesterase level, MMSE score, and total FIM score were combined, the area under the curve increased additively, suggesting that the prognostic risk of heart failure patients can be better evaluated.
Supplementary Table 3.
Supplementary log-log plots showed that the survival curves for each group scored by serum cholinesterase, MMSE score, and total FIM score did not intersect.
