# Supplementary material for: “Synergistic Effects of Nutritional, Cognitive, and Cardiac Rehabilitation Parameters on Prognosis in Patients with Systolic Heart Failure”
Source: JMA J. 2026 Aug 31;9(5):1310–21. doi: 10.31662/jmaj.2026-0022 (PMC13639507; doi:10.31662/jmaj.2026-0022)
Supplement: Supplementary file 1 [file 2433-3298-9-5-1310-s001.pdf]

Supplementary material Table.1. Net Reclassification Index (NRI)

(a) Results of logistic multivariate analysis

Variable	Multivariate analysis				
		95% CI			
	Odds Ratio	Lower	Upper	*p*-value	Reciprocal
NYHA functional class	5.113	2.933	9.856	< 0.0001	0.195
eGFR	0.984	0.975	0.992	0.0002	1.016
Serum cholinesterase	0.996	0.993	0.998	0.0086	1.004
MMSE score	0.922	0.864	0.983	0.0143	1.084
Total FIM score	0.963	0.953	0.971	< 0.0001	1.038

(b)

Model-1 NYHA + eGFR + ChE

Model-2 NYHA +eGFR + ChE +MMSE

Model-3 NYHA + eGFR + ChE + total FIM

Model-4 NYHA + eGFR + ChE + MMSE + total FIM

Model-1 vs. **Model-2**

**Model-1**	**Model-2**		
Subjects who had a cardiac event			
	≦ 25%	25 < and ≤ 50%	50% ≤
≤ 25%	19	4	5
25 < and ≤ 50%	27	40	53
50% ≤	0	19	136
Subjects who did not have a cardiac event			
	≦ 25%	25 < and ≤ 50%	50% ≤
≤ 25%	167	8	3
25 < and ≤ 50%	169	80	28
50% ≤	0	29	20

	**Improved**	**Made worse**	**Same**	**NRI (%)**	**p-value**
**Subjects who had a cardiac event**	62	46	195	5.3	p = 0.123
**Subjects who did not have a cardiac event**	198	16	290	-31.5	p < 0.0001
**Entire population**	260	62	485	36.8	p < 0.0001

Model-1 vs. **Model-3**

**Model-1**	**Model-3**		
Subjects who had a cardiac event			
	≦ 25%	25 < and ≤ 50%	50% ≤
≤ 25%	14	2	12
25 < and ≤ 50%	29	32	59
50% ≤	4	12	178
Subjects who did not have a cardiac event			
	≦ 25%	25 < and ≤ 50%	50% ≤
≤ 25%	168	8	2
25 < and ≤ 50%	201	50	26
50% ≤	5	28	16

	**Improved**	**Made worse**	**Same**	**NRI (%)**	**p-value**
**Subjects who had a cardiac event**	73	45	185	9.2	p = 0.009
**Subjects who did not have a cardiac event**	234	36	234	-39.2	p < 0.0001
**Entire population**	307	81	419	48.5	p < 0.0001

Model-2 vs. **Model-3**

**Model-2**	**Model-3**		
Subjects who had a cardiac event			
	≦ 25%	25 < and ≤ 50%	50% ≤
≤ 25%	30	7	10
25 < and ≤ 50%	10	29	23
50% ≤	4	12	178
Subjects who did not have a cardiac event			
	≦ 25%	25 < and ≤ 50%	50% ≤
≤ 25%	317	12	7
25 < and ≤ 50%	51	52	14
50% ≤	6	22	23

	**Improved**	**Made worse**	**Same**	**NRI (%)**	**p-value**
**Subjects who had a cardiac event**	40	26	237	4.6	p = 0.0848
**Subjects who did not have a cardiac event**	79	33	392	-9.1	p < 0.0001
**Entire population**	119	59	629	13.7	p < 0.0001

Model-2 vs. **Model-4**

**Model-2**	**Model-4**		
Subjects who had a cardiac event			
	≦ 25%	25 < and ≤ 50%	50% ≤
≤ 25%	34	10	3
25 < and ≤ 50%	9	36	17
50% ≤	1	12	181
Subjects who did not have a cardiac event			
	≦ 25%	25 < and ≤ 50%	50% ≤
≤ 25%	321	10	5
25 < and ≤ 50%	62	45	10
50% ≤	1	24	51

	**Improved**	**Made worse**	**Same**	**NRI (%)**	**p-value**
**Subjects who had a cardiac event**	30	13	260	5.6	p = 0.0095
**Subjects who did not have a cardiac event**	87	25	392	-12.3	p < 0.0001
**Entire population**	117	38	652	17.9	p < 0.0001

Model-3 vs. **Model-4**

**Model-3**	**Model-4**		
Subjects who had a cardiac event			
	≦ 25%	25 < and ≤ 50%	50% ≤
≤ 25%	40	4	0
25 < and ≤ 50%	4	50	4
50% ≤	0	4	207
Subjects who did not have a cardiac event			
	≦ 25%	25 < and ≤ 50%	50% ≤
≤ 25%	365	9	0
25 < and ≤ 50%	19	58	3
50% ≤	0	12	32

	**Improved**	**Made worse**	**Same**	**NRI (%)**	**p-value**
**Subjects who had a cardiac event**	8	8	287	0.0	p = 1.000
**Subjects who did not have a cardiac event**	31	12	467	-3.7	p = 0.0037
**Entire population**	33	20	754	3.7	p = 0.0419

Supplementary material Table 2. Incrementality of Area under the ROC Curve

(a) NYHA + eGFR + serum ChE

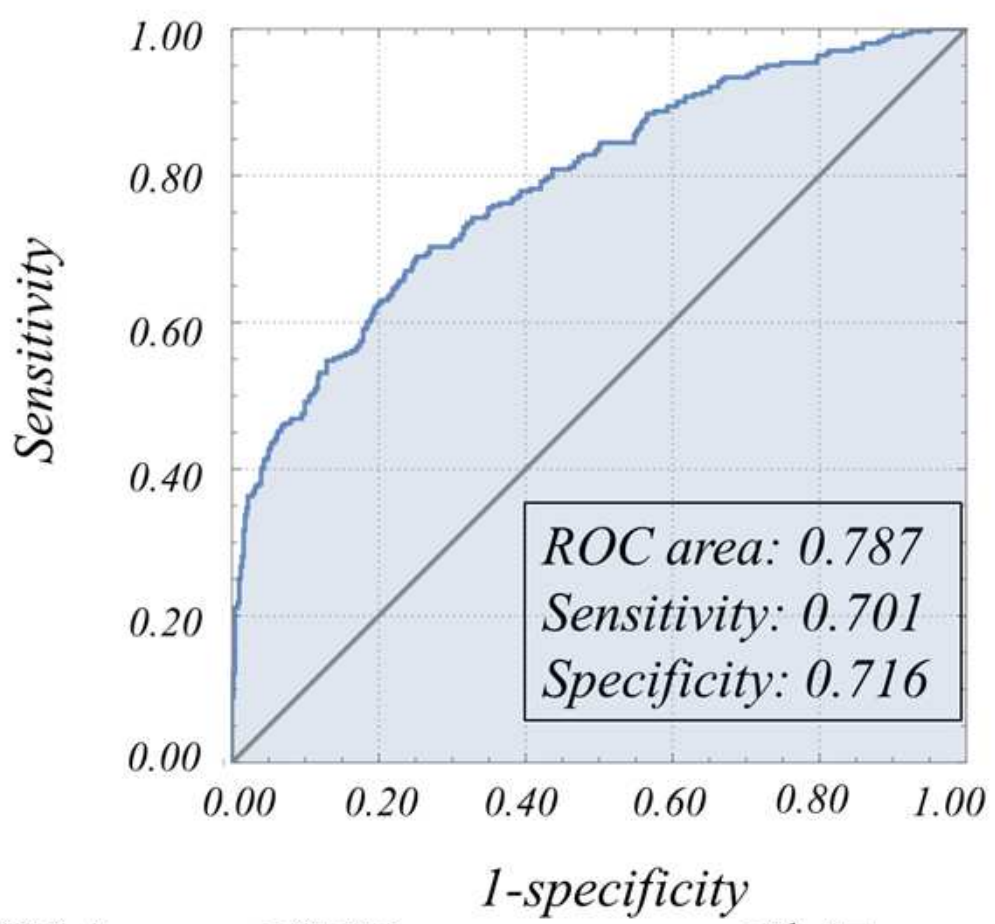

(b) NYHA + eGFR + serum ChE + MMSE score

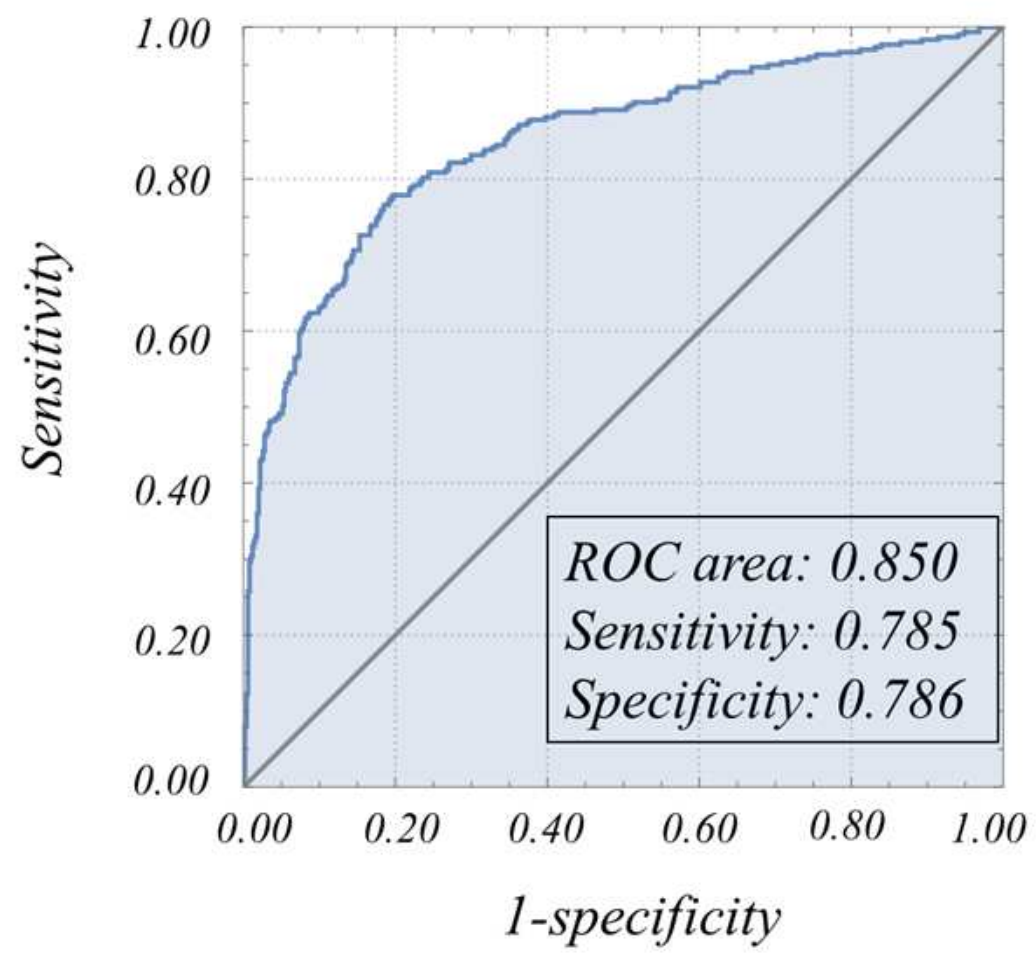

(c) NYHA + eGFR + serum ChE + total FIM score

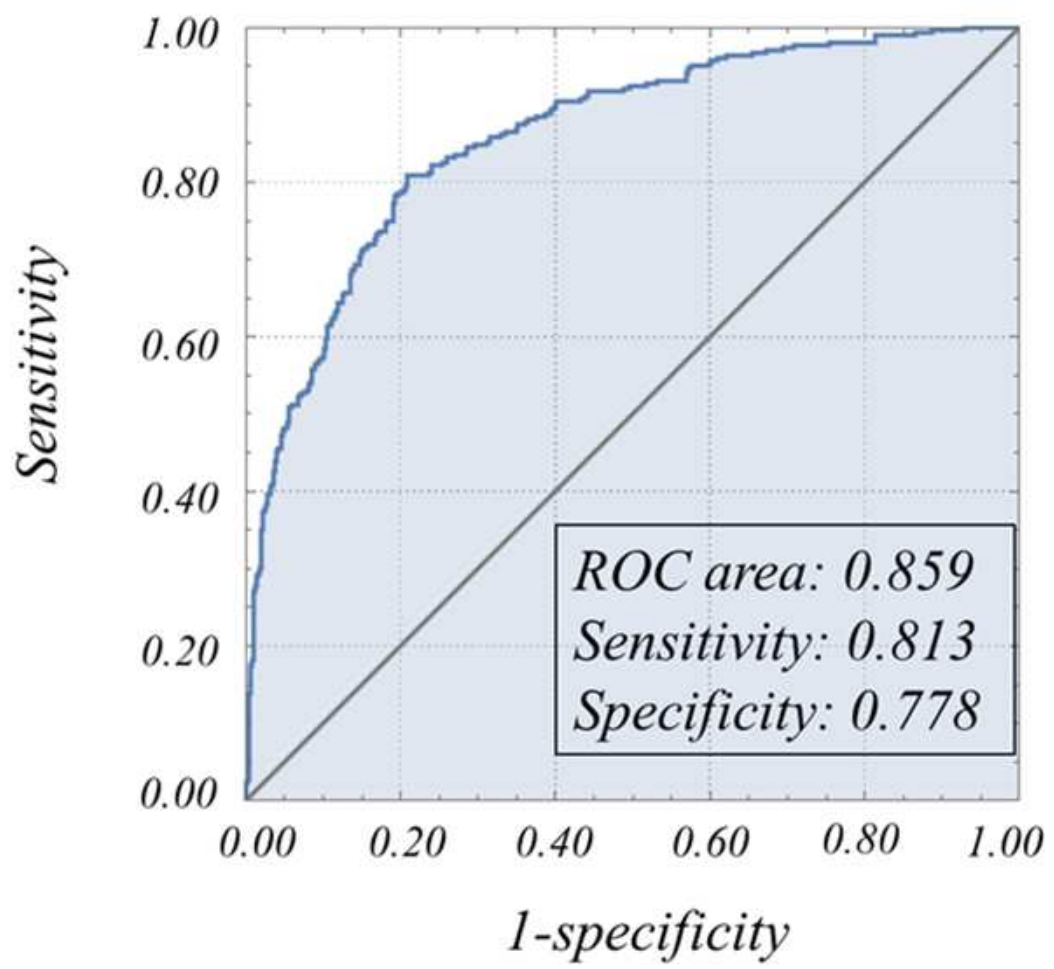

(d) NYHA + eGFR + serum ChE + MMSE score + total FIM score

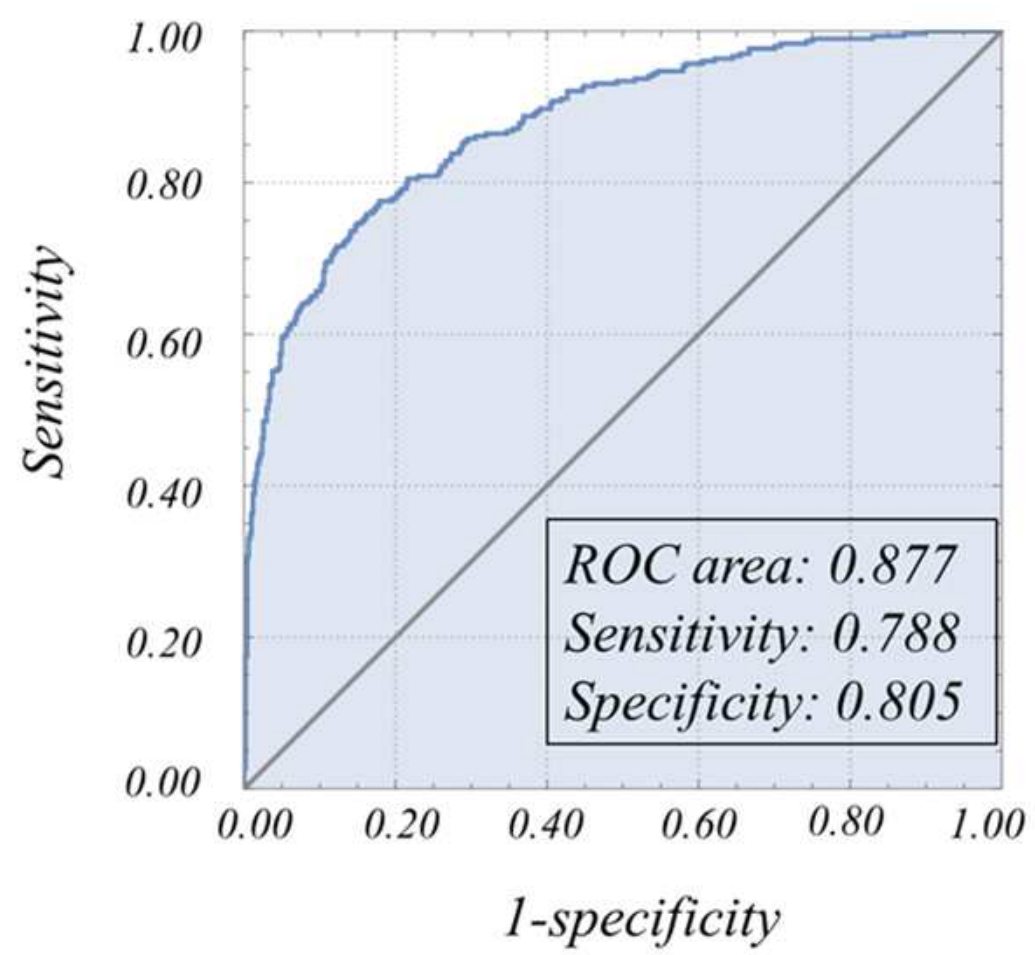

Supplementary material Table 3. Log-time Plot and Complementary Log-log Plot

(a)

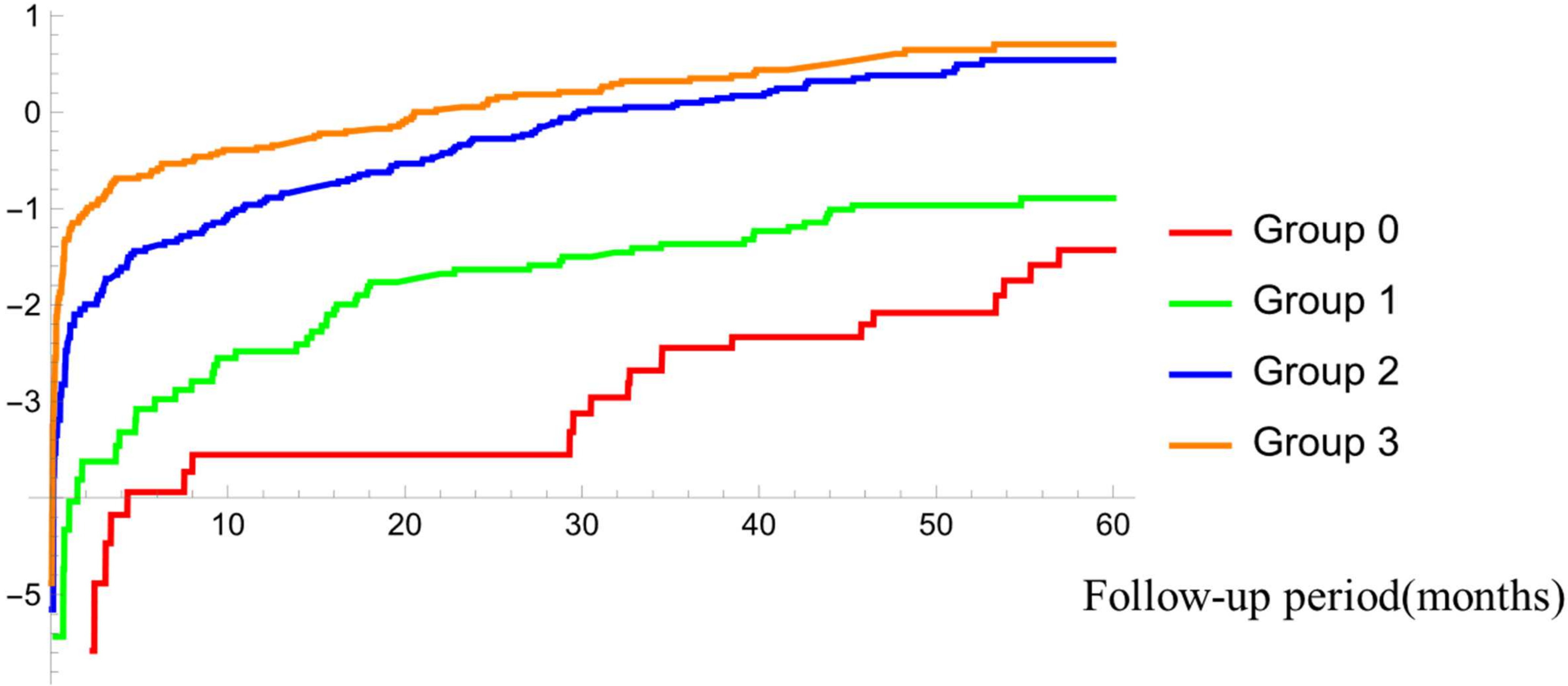

(b)

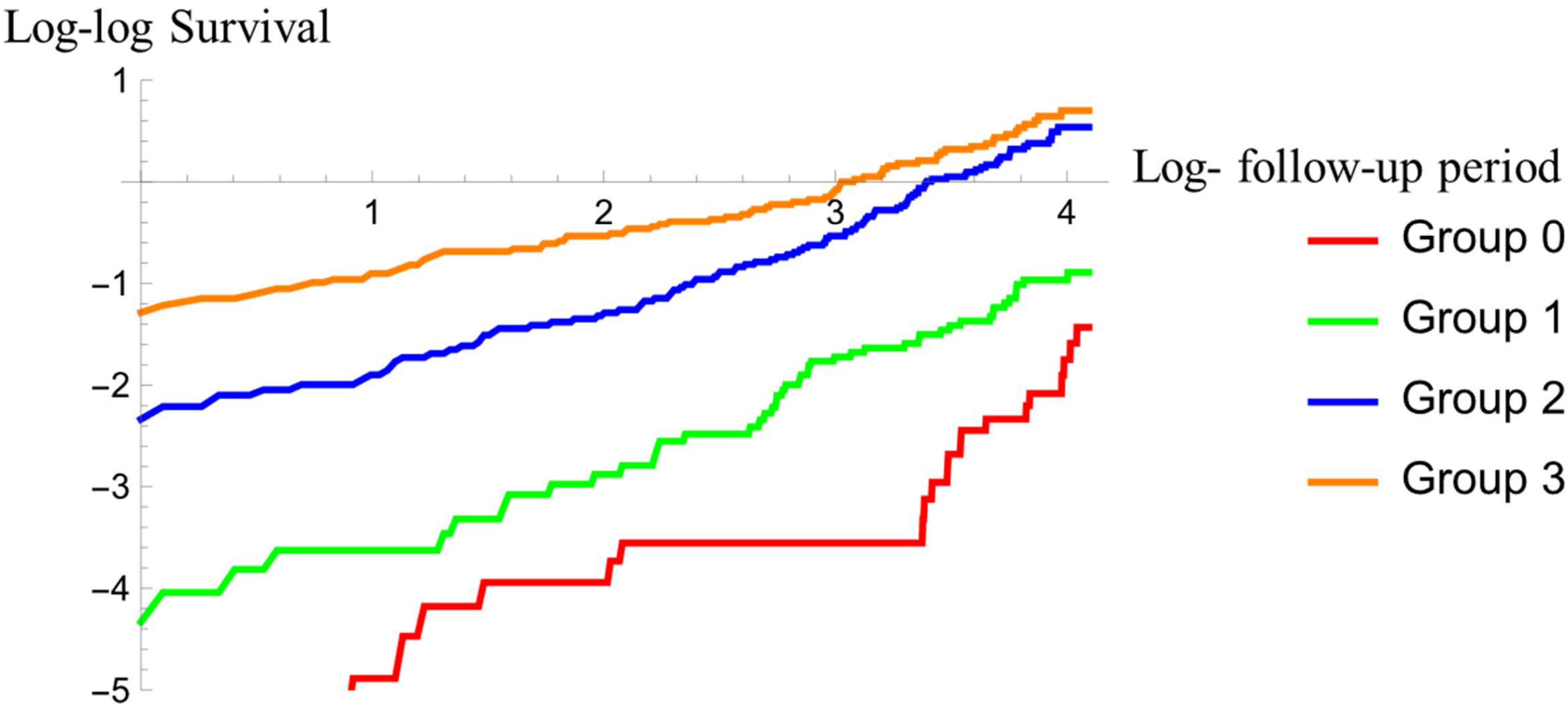

Log-log Survival
1
−1
−2
−3
−4
−5
1
2
3
4
Log- follow-up period
Group 0
Group 1
Group 2
Group 3
